# Novel Missense Variants in *TRIM37* Associated with Mulibrey Nanism and Complex Congenital Heart Disease

**DOI:** 10.26502/fccm.92920481

**Published:** 2026-03-03

**Authors:** Gloria K. E. Zodanu, Angela C. Zeigler, Jordan Mudery, Charlotte Wolf, John H. Hwang, Xuedong Kang, Amy Speirs, Lee-Kai Wang, Reshma Biniwale, Ming-Sing Si, Nancy Halnon, Gary M. Satou, UCLA Congenital Heart Defects BioCore Faculty, Wayne W. Grody, Glen S. Van Arsdell, Stanly F. Nelson, Marlin Touma

**Affiliations:** 1 Neonatal Congenital Heart Laboratory, Department of Pediatrics, David Geffen School of Medicine, University of California, Los Angeles, CA, USA; 2 Department of Pediatrics, David Geffen School of Medicine, University of California, Los Angeles, CA, USA; 3 Department of Human Genetics, David Geffen School of Medicine, University of California, Los Angeles, CA, USA; 4 Department of Surgery, David Geffen School of Medicine, University of California, Los Angeles, CA, USA; 5 Department of Pathology and Laboratory Medicine, David Geffen School of Medicine, University of California, Los Angeles, CA, USA; 6 Molecular Biology Institute, University of California, Los Angeles, CA, USA; 7 Children’s Discovery and Innovation Institute, University of California, Los Angeles, CA, USA; 8 Eli and Edyth Broad Stem Cell Research Center, University of California, Los Angeles, CA, US; 9 Cardiovascular Research Laboratories, David Geffen School of Medicine, University of California, Los Angeles, CA, USA

**Keywords:** Mulibrey nanism, *TRIM37*, Whole Exome Sequencing (WES), Genetic variants

## Abstract

**Background:**

Mulibrey nanism is a rare autosomal recessive genetic disorder caused by homozygous or compound heterozygous mutations in the tripartite motif protein 37 (*TRIM37* gene), which codes for a RING finger E3 ubiquitin ligase. Affected individuals present with prenatal-onset growth failure, abnormalities in multiple organs, and heart disorders, including constrictive pericarditis and restrictive cardiomyopathy.

**Methods and Results:**

We performed phenotypic-genotypic analyses in a 32-week gestation preterm infant presenting with a complex congenital heart disease, including interrupted aortic arch type B, persistent left-sided superior vena cava (SVC), septal defects, and valvular disease, in addition to other congenital malformations. Initial genetic screenings, including whole genome chromosomal microarray, were negative. Whole exome sequencing (WES) of DNA samples from the proband/mother duo revealed two novel heterozygous missense variants [c.199C>T (p.Arg67Cys)] and [c.2041C>G (p.Gln681Glu)] in the *TRIM37* gene, which has been associated with Mulibrey nanism. The c.199C>T variant was inherited from the mother; however, the mode of inheritance of the c.2041C>G could not be ascertained. Whether the two variants are present on the same allele could not be determined either, due to the unavailability of the father or any family member for additional genetic analysis. However, *in silico* analysis predicted that both variants are damaging, and both amino acid positions are fairly well conserved.

**Conclusion:**

These findings highlight the critical role of the *TRIM37* genetic variants in complex congenital heart defect phenotypes associated with Mulibrey nanism and emphasize the importance of comprehensive triobased WES for antenatal care and early diagnosis.

## Introduction

Mulibrey nanism, or MN (OMIM 253250), is a very uncommon, severe genetic disorder that was first reported by Perheentupa et al. [1] in 1970, in a study of four Finnish patients [1,2]. The disorder was named Mulibrey nanism, with the Mulibrey term created from the names of the organs mainly involved MUscle, LIver, BRain, Eye [2]. Nanism describes the short stature (dwarfism) that is frequently seen in individuals with this disorder [2]. In the earlier report by Perheentupa, they also identified twenty-three cases of Mulibrey nanism in patients between 4 months and 23 years with progressive growth failure and congestive heart failure. Mostly, constrictive pericarditis was the main cardiac feature that was distinct at birth [1,2]. Other clinical features observed in some cases include myocardial hypertrophy, myocardial fibrosis, muscular hypotonia, triangular face, voice changes, pigmented fundi, hepatomegaly, cutaneous nevi flammae, fibrous dysplasia of long bones, elevated jugular venous pressure, lack of sexual development, insulin resistance and type 2 diabetes, and an increased risk for tumours [3–8]. Underdevelopment of the sex organs is mostly not seen in MN, but females with the disorder are usually infertile and tend to develop ovarian failure before 30 years of age [9]. The disorder is caused by a mutation in the *TRIM37* gene that encodes a tripartite motif (TRIM) family protein [5,8]. This affects numerous mesodermal tissues. Mulibrey nanism is typically inherited in an autosomal recessive pattern. Hence, a single heterozygous variant (being a carrier) does not typically cause the disease. Instead, the disease arises from compound heterozygous mutations, which result in the inheritance of two distinct yet mutated, alleles—inherited from each parent. Although it seems quite rare, Mulibrey nanism is relatively more prevalent in the Finnish population [3,10]. As of 2019, 140 patients from Finland had been identified, suggestive of a founder effect. Isolated, infrequent cases have also been reported from different populations worldwide [3,10]. It is noteworthy that the clinical features of this disease are quite diverse and may intersect and overlap with other conditions, including Silver-Russell syndrome [6].

### The TRIpartite Motif (TRIM) protein family

**Figure 1:**
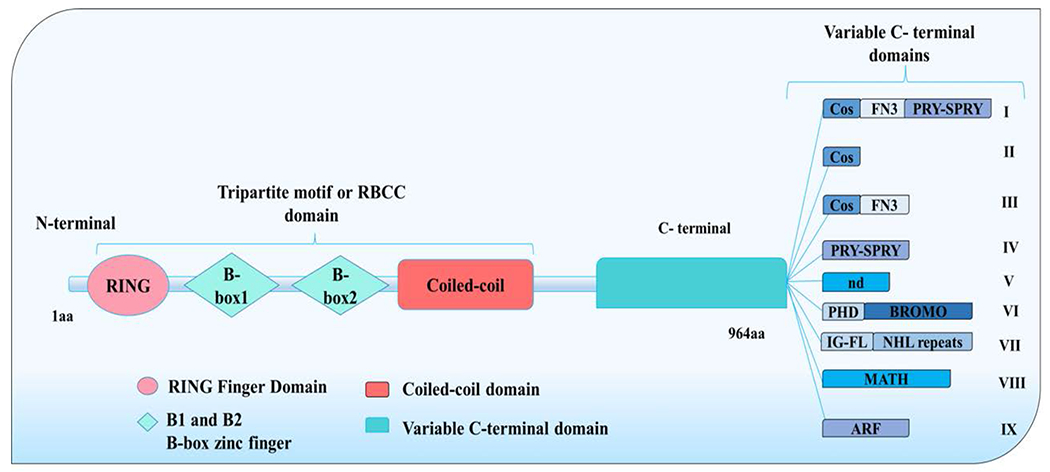
Domain structure and function of the TRIM protein. The TRIM family organization at the N-terminal including a TRIM motif or RBCC (Ring-B-box-Coiled-coil) domain, and the variable C-terminal domain sub-classified into nine subgroups (class I-IX). TRIM37 is a member of class VIII. Adopted from Meroni (2020) [8].

The TRIM protein is part of a novel family of zinc finger proteins [7,11] that was first discovered by Reymond et al. in 2001 by studying 37 TRIM genes/proteins using functional genomic approaches [12]. He demonstrated that the TRIM motif has a RING domain, one or two B-box motifs, and a coiled-coil domain, with similar biological functions at the amino terminal (N-terminal) [6]; hence, it was previously known as the RBCC for Ring-B-box- Coiled-coil [6]. All TRIMs have the same N-terminal tripartite domain arrangement, RBCC, but differ in their carboxyl-terminal (C-terminal) domains [13] (Figure 1). In most cases, TRIM proteins act as E3 ubiquitin ligases and are responsible for ensuring an appropriate and accurate transfer of the ubiquitin components to target sites [8,14]. Following the N-terminal is the variable C-terminal domain, which has diverse domains that make it possible to be classified into nine subgroups that interact with specific target proteins, resulting in their distinct roles [13,15]. Representation of these domains is based on Meroni (2020) [8] as seen in Figure 1.

### Classes of the TRIM family

TRIM family class I is characterized by the existence of both B-box 1 and B-box 2 in the tripartite motif, in addition to a COS domain, followed by a fibronectin type III repeat (FN3) and a PRY-SPRY domain at the C- terminal domain (Figure 1). The PRY (pyrin) domain has about 61 amino acids, and the SPRY is made of 160 amino acid residues that are determined based on the organism and protein. The PRY-SPRY domain is present in more than 500 proteins involved in proliferation, innate immune response, and cytokines signaling [6]. Class I members are often expressed during the embryonic stages. Consequently, developmental disorders in children are linked with abnormalities in some of these TRIM members, including TRIM18, which causes the X-linked form of Opitz G/BBB Syndrome [8]. Class VII members have both B-box-1 and B-box-2 within the tripartite motif, whereas some members of this same group only have the B-box-2 domain. Their C-terminal can be distinguished by the occurrence of a 6-bladed β propeller-like structure made up of 6 NHL (NCL-1, HT2A, LIN41) repeat (Figure 1). However, some also have a Filamin-type immunoglobulin domain (IGFLMN). A genetic mutation in the *TRIM27* gene is reported to cause Charcot-Marie-Tooth disease [8]. The other classes of TRIM are linked with several genetic disorders, such as class VIII, which includes TRIM37 that has been linked with Mulibrey nanism [8]. Currently, over 100 TRIM members have been identified as key players in several cellular activities, including apoptosis, differentiation, proliferation, transcription, DNA repair, and regulation of immune and cell stress response [13,16]. Here, we conduct comprehensive phenope-genotype analyses on a preterm infant with two novel heterozygous variants in the *TRIM37* gene, who presented with severe congenital malformations suggestive of Mulibrey nanism, associated with a complex congenital heart defect phenotype and a lethal prognosis.

## Materials and Methods

### Human Studies

All human studies were conducted in accordance with the regulations of the University of California, Los Angeles (UCLA) Institutional Review Board (IRB). Subjects provided written informed consents to participate in this study. Electronic medical records, family history, and specimen collection were acquired through the UCLA Congenital Heart Defect (CHD)-BioCore following the UCLA-IRB-approved protocols. Samples were de-identified and coded following acquisition. Prenatal diagnosis was determined based on fetal sonography findings. Clinical diagnosis was determined based on clinical features, sonography, magnetic resonance imaging (MRI), and echocardiography findings.

### Cytogenomic SNP Microarray

SNP Microarray was performed using genomic DNA (gDNA) isolated from peripheral blood monocytes at the UCLA Clinical Genomics Center following clinically validated CLIA (Clinical Laboratory Improvement Amendments)- and CAP (College of American Pathologists)-validated protocols. A whole-genome single- nucleotide polymorphism (SNP) oligonucleotide array was used to assess copy number variations (CNVs), insertions, deletions, duplications, and genomic imbalances in the sample tested. The assay compared the proband’s DNA to an internal reference and to an external reference from 380 normal controls using the Affymetrix Genome-Wide SNP Array CytoScan^™^ HD (ThermoFisher Scientific, Waltham, MA, USA) for both normalization and comparative analysis. This array platform contains 2.6 million markers for CNV detection, of which 750,000 are genotype SNPs and 1.9 million are non-polymorphic probes, for whole-genome coverage. The analysis was performed using the Affymetrix Chromosome Analysis Suite (ChAS) software, version 3. 1.0.15 (r9069). The assay was designed to detect alterations to copy number errors (loss or gain) as well as copy-neutral alterations (regions of homozygosity) that indicate an absence or loss of heterozygosity.

### Whole-Exome Sequencing (WES)

The genomic DNA was extracted from peripheral blood monocytes (PBMCs) at the UCLA Congenital Heart Defects BioCore by using standard methods (Purelink Genomic DNA Mini Kit, Invitrogen, Waltham, MA, USA). Library preparation, sequencing, and data analysis were performed at the CCRD (California Center for Rare Disease) and the UCLA Clinical Genomics Center, using the CLIA-validated protocols. Genomic DNA (3 μg) samples from the proband/mother duo were subjected to library preparation and exome capture following the Agilent Sure Select Human All Exon 50 Mb (Agilent Technologies, Santa Clara, CA, USA) Illumina Paired-End Sequencing Library Prep Protocol. Sequencing was performed on an Illumina HiSeq4000 (Illumina, San Diego, California, United States) as a 50 bp paired-end run. For each sample, approximately 200 million independent paired reads were generated for an average coverage of 140X of RefSeq protein-coding exons and flanking introns (+/− 2 bp), with at least 99% of these bases covered at ≥10×. The data showed a high-quality genomic sequence meeting a stringent human genomic variation quality standard. The sequences were aligned to the hg19/b37 genome release by using the Novoalign function. PCR duplicates were marked using Picard. The Genome Analysis Toolkit (GATK)33 was used for indel realignment and base quality recalibration. Both SNVs (single- nucleotide variants) and small INDELs (insertions and deletions) were called using GATK unified genotyper. All variants were annotated using the customized VEP (variant effect predictor) engine from Ensembl. Regions of homozygosity by descent were determined using PLINK. Rare variants with a minor allele frequency of <1% in public databases were retained for further analysis.

### Variant Analysis

Candidate rare variants were classified based on their zygosity and pattern of inheritance, their location within the gene, conservation scores, population and allele frequencies (ClinVar), predicted consequence at the protein level and structural domains, pathogenicity prediction in silico tools, evidence from functional studies and animal models, and disease spectrum, in accordance with the ACMG/AMP Guidelines for Interpretation of Sequence Variants [34]. All variants were interpreted in the context of the patient’s phenotype. Variants were dismissed if they were predicted to be tolerant (have low impact on protein structure or function) or have been reported in the GnomAD database. Finally, the technical quality of the candidate variants was confirmed using the Integrative Genomics Viewer (IGV) v2.16.0 [35]. Only significant variants that are associated with the primary clinical concern(s) were reported. Variants of uncertain significance, which are unrelated to the primary clinical concern(s) as well as any incidental findings that are not deemed to be pertinent, were not reported.

## Results

### History and Clinical Course

**Figure 2:**
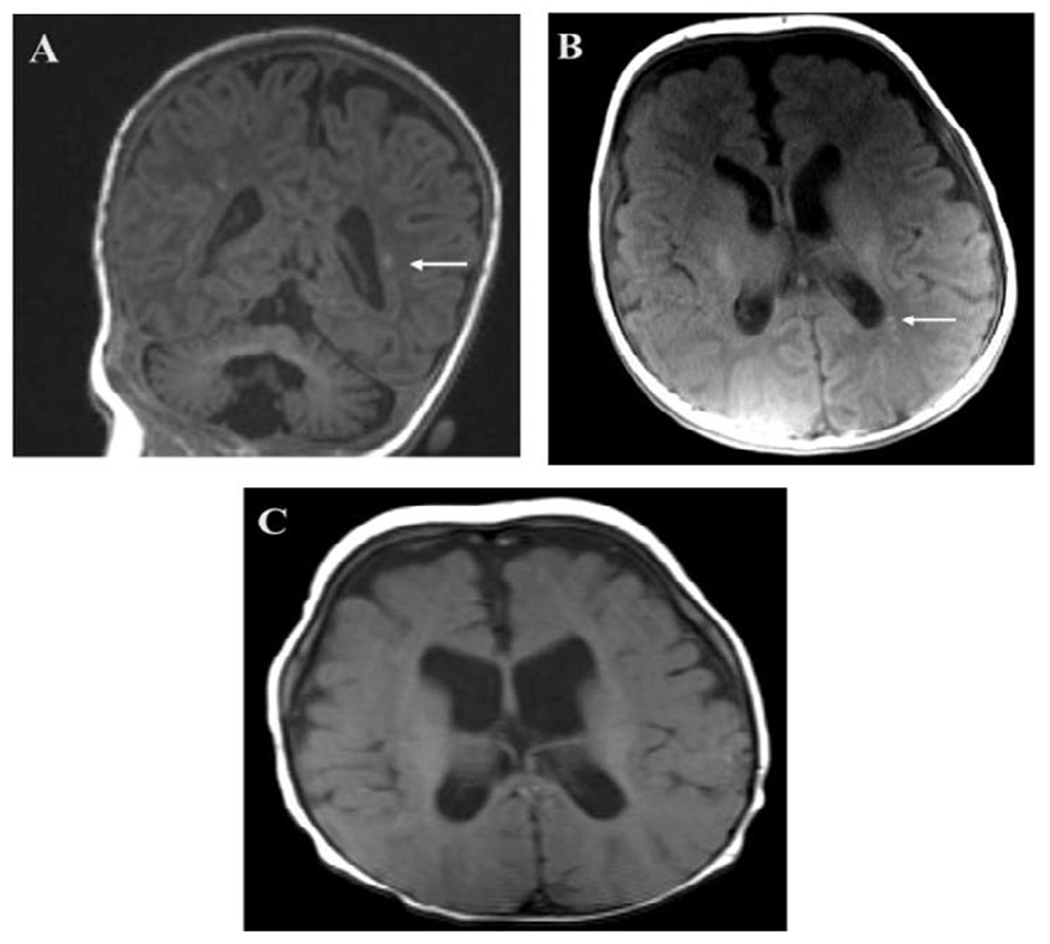
Representative brain MRI showing progressive periventricular leukomalacia (PVL). T1 images from scan at 2 months of age (A and B) showing areas of hyperintensity suggesting early PVL marked with white arrows. T1 image from follow up scan at 5 months of age showing ventricular enlargement compared to prior and ex vacuo of the periventricular white matter as evidence of late stage PVL.

The proband is a male premature infant who was born at 32 weeks-gestation. This was the first pregnancy of a 30-year-old healthy mother with poor prenatal care, who presented in labor. Limited fetal ultrasound showed fetal growth restriction, oligohydramnios, and a small right polycystic kidney. Due to non-reassuring fetal heart tracings, an urgent cesarean delivery was conducted. Upon delivery, an omphalocele and cleft lip and palate were noted. The infant was intubated due to poor respiratory effort. After initial stabilization, the infant was admitted to a tertiary care facility for intensive neonatal care and omphalocele repair. Throughout the course, the infant remained intubated, requiring continuous mechanical ventilation. Head ultrasound (HUS) showed mild prominence of the lateral ventricles and extra-axial spaces. Serial brain imaging showed focal coagulation necrosis in the white matter, evolving periventricular leukomalacia, and subsequent progressive ventriculomegaly due to white matter volume loss (Figure 2). Electroencephalogram recordings showed no seizure activity.

**Figure 3:**
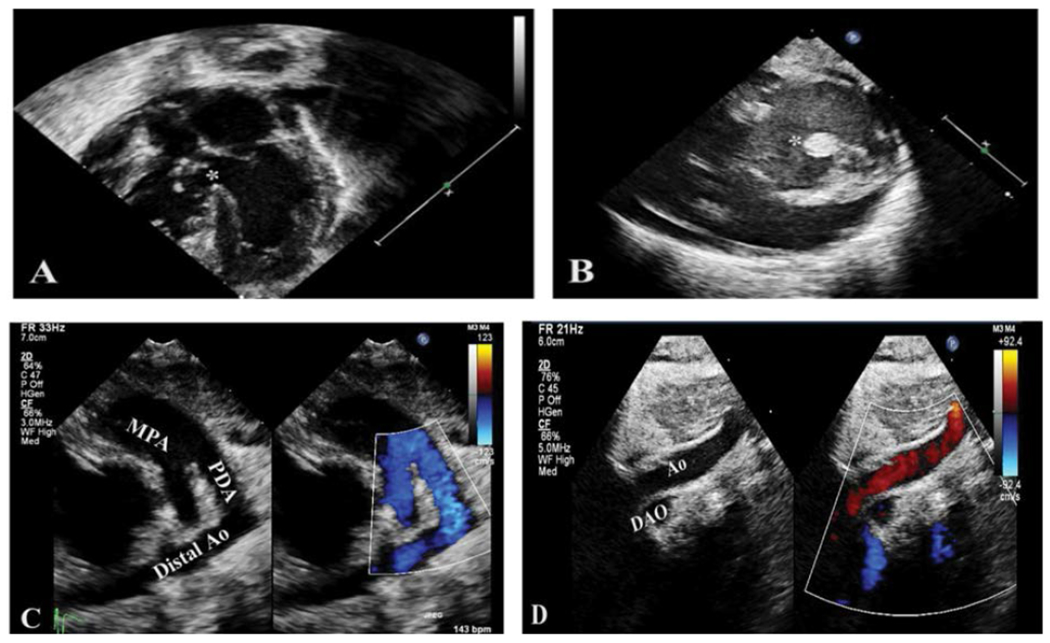
Representative echocardiography images of proband showing multiple cardiac malformations. (A) 5-chamber view showing VSD and posterior malaligned conal septum (asterisk); (B) Parasternal short axis view showing VSD (asterisk); (C) Color Doppler interrogation of the ductus arteriosis (PDA) showing origin at the main pulmonary artery (MPA) and ductal arch with unobstructed right to left flow to the distal aorta (Distal Ao). (D) Suprasternal notch view of aortic arch (Ao) demonstrating interruption without communication with the descending aorta (DAO).

**Figure 4:**
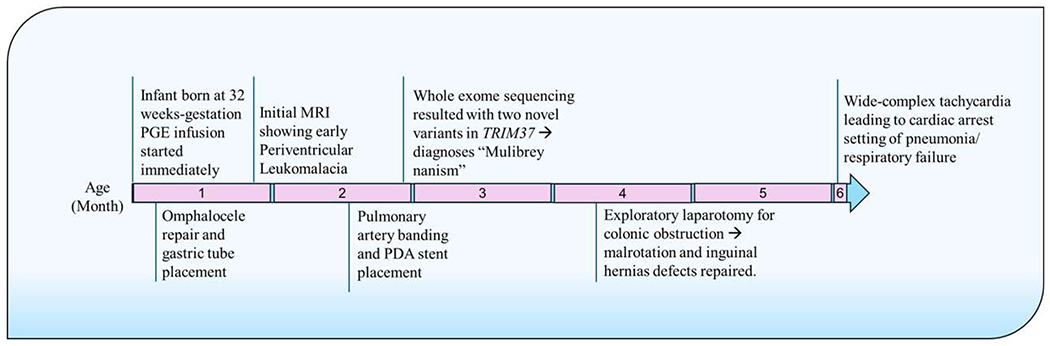
Timeline of major clinical events.

Ophthalmological assessment showed iris hypoplasia, but with normal vascularization of the retina in both eyes. Abdominal ultrasound showed a distorted right adrenal gland and a right solitary multidysplastic kidney with right hydroureteronephrosis. Cardiovascular evaluation revealed complex critical congenital cardiac anomalies (Figure 3), including, interrupted aortic arch type B, right ventricle (RV) dilation, tricuspid valve dysplasia with moderate insufficiency and right atrial enlargement, moderate-sized peri-membranous ventricular septal defect (VSD), large patent ductus arteriosus (PDA), persistent left-sided superior vena cava (SVC) draining into the coronary sinus, mildly small aortic valve annulus, and moderate RV hypertrophy. The infant received prostaglandin E1 (PGE1) infusion initially, followed by stent placement to maintain ductal patency. Given the dismal prognosis with the high likelihood of death versus survival with severe impairments, no additional cardiac surgeries were offered. The mother received extensive counseling regarding the poor prognosis, but she desired to continue intensive medical management for the infant. The subsequent course was complicated by chronic respiratory insufficiency, direct hyperproteinemia, and necrotizing enterocolitis that required exploratory laparotomy. The infant’s clinical status continued to deteriorate gradually with multiorgan failure. He subsequently died at six months of age due to cardiorespiratory failure with a wide-complex tachycardia episode that did not respond to resuscitation efforts leading to cardiac arrest (Figure 4).

The family history is unremarkable, with no history of congenital heart defects or other congenital anomalies. Both parents and other immediate family members are healthy. No history of consanguinity. No history of exposure to biohazardous substances or tobacco.

### Genetic Workup

#### Karyotype and Fluorescence in situ hybridization (FISH) analysis

FISH for Trisomy 13, 18, and 21 showed negative results, and karyotype analysis showed normal chromosomes, 46 XY.

#### Cytogenetic Whole-Genome SNP Microarray

Normal chromosomal arrays were observed with no copy number changes.

#### Duo Whole-Exome Sequencing (WES)

Duo WES was performed using isolated DNA samples from peripheral blood monocytes (PBMC) of the proband and his mother (the father was not available). The proband’s WES data analysis was consistent with 46, XY male, with no increased regions of homozygosity (>5Mb). Exome variant analysis was performed in accordance with American College of Medical Genetics and Genomics (ACMG) recommendations. A primary gene list was generated from the Human Gene Mutation Database (HGMD) and Online Mendelian Inheritance in Man (OMIM) using the keywords congenital heart defect, interrupted aortic arch, VSD, dilated RV, or PDA, as well as dysplastic multisystem kidney, cleft palate, and omphalocele. Within the primary gene list and the genes annotated, there were no established clinically significant variants, single-nucleotide polymorphisms (SNPs), or small deletions or insertions (<10bp) identified that could explain the primary clinical concerns seen in our patient. However, two novel heterozygous variants [chr17:g.57165734C>T (p.Arg67Cys)] and [chr17:g.57105992C>G(p.Gln681Glu)] of uncertain clinical significance were identified in the *TRIM37* gene. Corresponding nucleotide changes (c.199C>T:c.2041C>G) on transcript NM_015294.3 are shown in Table 1. The variant call quality was high for both variants.

## Discussion

### Variants Interpretation

**Figure 5:**
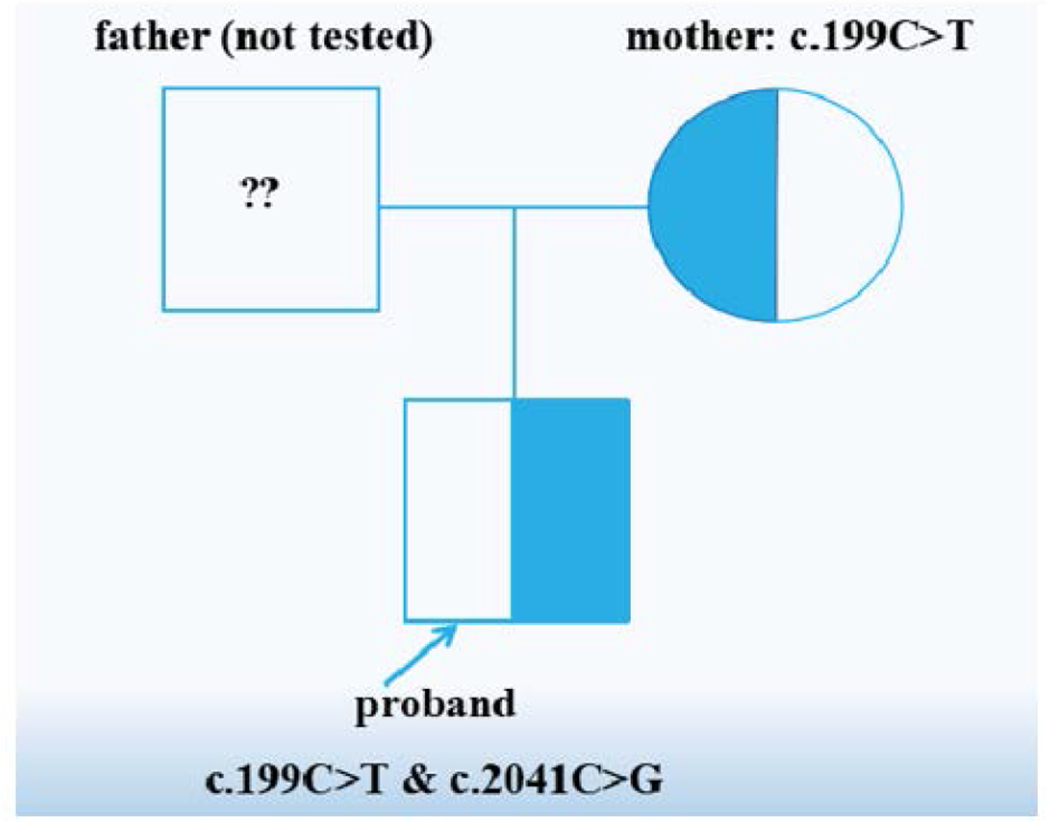
Family pedigree of proband.

We conducted clinical phenotyping and genomic analysis in a premature male infant (proband) of nonconsanguineous parents. His phenotype included complex critical congenital heart defects, including interrupted aortic arch Type B, peri-membranous VSD, and RV dilation and hypertrophy, in addition to severe congenital anomalies involving the palate and facial structures, the right kidney, the central nervous system, and the abdominal wall. Genomic analysis revealed two novel heterozygous variants [c.199C>T (p.Arg67Cys) and c.2041C>G (p.Gln681Glu)] in the *TRIM37* gene, which is a gene associated with Mulibrey nanism (Table 1, Figure 5).

The c.199C>T variant was inherited from the mother. In the corresponding protein, at position 67 of the amino acid, the arginine (A) is replaced with a cysteine (C) residue on the negative strand. The c.2041C>G variant was not detected in the mother. In the corresponding protein, at position 681 of the amino acid, the glutamine (C) is replaced with glutamic acid (G), also located on the negative strand. Neither of these variants has been observed in the general population nor reported in the literature in individuals with this condition. However, *in silico* analysis using PolyPhen-2 and SIFT predicted that both variants are damaging, and both amino acid positions are well conserved. Interestingly, whether these two variants are present on the same allele or opposing alleles cannot currently be ascertained. Even though testing of the father and other family members was recommended to assess the phase of any potentially compound heterozygous variant, no sample was submitted.

### *TRIM37* Gene and Functional TRIM37 Protein Domains

**Figure 6:**
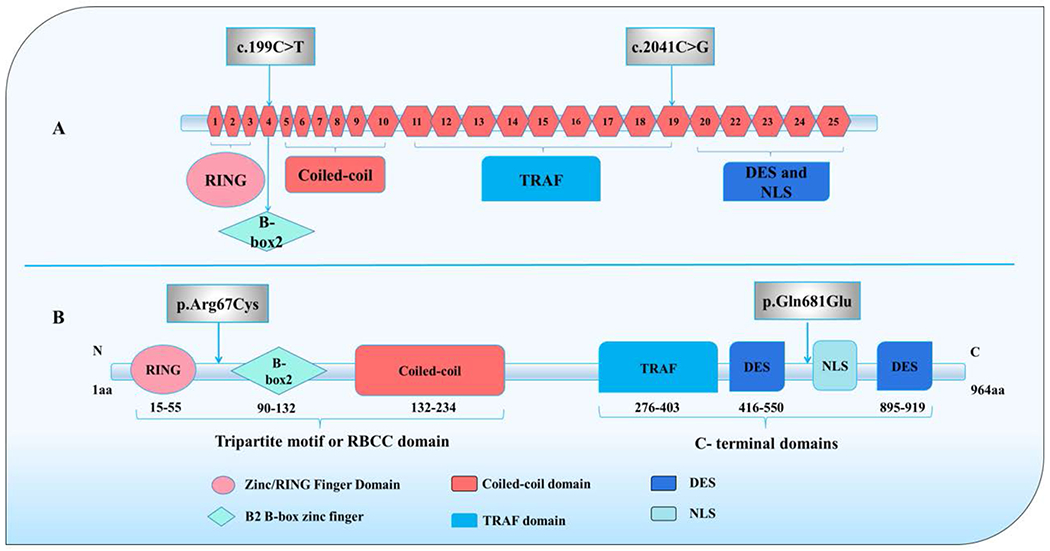
Schematic diagram of the *TRIM37* gene and encoded protein showing the position of the detected variants in the proband. (**A**) Schematic diagram showing the exons, their corresponding domains, and position of the novel missense variants in the *TRIM37* gene in our proband. (**B**) Schematic diagram showing the domains of the TRIM37 protein and the positions of the corresponding amino acid substitutions due to the novel missense variants detected in our proband.

The human *TRIM37* gene is located on chromosome 17q22-q23, spanning twenty five exons (Figure 6A) [5,6,17]. The encoded TRIM37 protein consists of 964 amino acids with a molecular weight of 108-130 kDa [6,18], and is expressed in several tissues and cellular organelles [3,5,10,19], with specific localization in the peroxisome [20], which is the cytoplasmic organelle that plays a role in lipid metabolism. Hence, in some cases, Mulibrey nanism is classified as a peroxisomal disorder due to the similarity in the features [20,21]. As a member of the tripartite motif (TRIM) family [3,8,18,22,23], the TRIM37 protein acts as an E3 ubiquitin-protein ligase, which is a type of enzyme that is responsible for the attachment of ubiquitin molecules to substrate proteins for degradation [3,8,14].

The N-terminal of the TRIM37 protein is occupied by the TRIM or RBCC domain, which comprises the RING domain (amino acids 5-55), the B-box domain (amino acids 90-132), a coiled-coil domain (amino acids 132-254), and the TRIM domain, and performs many functions of the protein (Figure 6 B) [3,8,18,22,23]. For instance, the RING domain, which is a zinc-binding domain, is essential for the binding of other proteins and plays a role in E3 ubiquitin ligase activity (posttranslational modification) [7,11]. The B-box domain has two types of B-box family, B- box1 and B-box2, and both have similar but diverse arrangements of cysteine and histidine residues that associate with the RING finger to promote its crucial role in protein ubiquitination [3,8,14]. B-box domains are found in diverse sub-cellular localization and are associated with various processes, including cellular proliferation [5]. The TRIM37 only has the B-box-2 domain. Lastly, the coiled-coil domain on the N-terminal has about 100 residues, which are mostly separated into two or three separate coiled-coil motifs. The main function of this domain is to enhance interaction between proteins and oligomerization, which is necessary for biological processes [3,6,8,14]. On the other end of the TRIM37 protein is the C-terminal domain (carboxyl domain), which is different for each TRIM family (Figure 6B) [13]. Uniquely, the C-terminal domain of TRIM37 contains a TRAF (tumor necrosis factor-receptor- associated factor) domain. Hence, TRIM37 was initially included in the TRAF family of proteins. This domain is necessary for the binding of TRAF proteins with other proteins, serving as a support molecule for receptors, kinases, and other regulators in signaling pathways [19]. Additionally, there are two DES (aspartate-glutamate- serine)-rich sequence domains (amino acids 416-550 and 895-919, respectively) that contain numerous aspartate, glutamate, and serine residues that share limited similarities with transcriptional regulators [3,8,18,22,23]. The NLS (nuclear localization sequence) domain directs the protein to the nucleus (Figure 6B) [3,8,18,22,23]. Despite our understanding of the functional components of this protein, the pathogenic pathways linking TRIM37 deficiency to Mulibrey nanism and associated heart disease or structural defects remain poorly defined [18].

### Novel TRIM37 variants and clinical presentation in the Proband

Compared to previously reported Mulibrey nanism cases, our case is distinct as highlighted below. At the phenotypic level, although the proband met the clinical constellation of Mulibrey nanism, he presented with several congenital malformations that are more severe compared to other Mulibrey nanism cases, signifying multiorgan manifestations that are distinct from previously reported cases in the literature.

The proband was born with complex and critical congenital heart defects involving the endocardial cushions (VSD, ASD), great vessels (aortic arch, left superior vena cava), and to a lower degree the valves (mitral valve, tricuspid valve). These congenital defects are markedly different from the cardiac phenotype commonly seen in Mulibrey nanism, which is typically comprised of constrictive pericarditis and restrictive cardiomyopathy that present early after birth and are typically the cause of death when present (Table 2) [1,4,24]. With the increasing number of patients identified with cardiac manifestations, the pathogenic variants have been restricted to the RBCC domain (RING-B-box-Coiled-coil zinc protein) of TRIM37, expressed in the peroxisomes and centromeres [5,17,19,25]. Hence, genetic studies have characterized Mulibrey nanism as a peroxisomal disorder [24]. Peroxisomes are subcellular organelles that play a crucial role in numerous cellular activities, including the breakdown of fatty acids and the synthesis of certain lipids. Studies have implicated that genetic variations in the transcriptional control of peroxisomal and mitochondrial fatty acid oxidation may lead to the development of left ventricular hypertrophy and several other diseases [24]. As affected individuals age, their quality of life is affected by the extent to which the pericarditis constricts heart function, resulting in congestive heart failure, which is the main cause of death in Mulibrey nanism [1,7,24]. Mechanistically, The role of ubiquitin during the developmental stages is a crucial functional property of mammals. Several processes, including cell division, apoptosis, migration, and differentiation, must be carefully regulated by ubiquitination for normal growth to occur [26]. As such, when there is a defect in the TRIM domain of TRIM37, it inhibits the normal functioning of the protein and affects the efficiency of cell division, resulting in several anomalies [26]. However, none of the known effects of mutations in *TRIM37* have a clear link to the congenital heart disease seen in our patient. In fact, our patient had several structural congenital heart defects, which were more severe compared to what has been previously reported in other cases of Mulibrey nanism, which could indicate a unique pathogenic mechanism for the variants identified. To our knowledge, there is no publication related to this phenotypic variant in the literature to date, and the otherwise negative, exome sequencing results do not readily suggest any modifier genes. Elucidating the exact mechanism of the complex congenital cardiac malformation in our proband would require developmental analysis in transgenic mice and confirmatory mechanistic studies.

At the genetic level, two novel variants in the *TRIM37* gene were detected in the proband. The pathogenicity of these variants is uncertain based on current knowledge. However, we can make some hypotheses about these variants based on the resulting biochemical, chemical, and structural changes in the TRIM37 protein. The novel variant [c.199C>T (p.Arg67Cys)] resulted in an arginine to cysteine substitution at position 67 on the negative strand, resulting in a missense mutation in exon 4 (Figure 6A). It could be that the location of this variant in the TRIM domain of the TRIM37 protein, and the huge size of the arginine compared to cysteine, which is small, interrupts the TRIM domain (Figure 6B). Consequently, the substitution from arginine, a positively charged, basic amino acid, to cysteine, a neutral sulfur-containing amino acid, may have changed the overall charge of the protein. This effect renders it unstable for other proteins to bind to the RING finger domain of TRIM37, thereby affecting the protein’s configuration and function. Alternatively, introduction of an aberrant cysteine residue could alter the protein’s 3-dimensional structure due to abnormal disulfide bonding. The second novel variant, c.2041C>G (p.Gln681Glu), resulted in a glutamine residue being replaced with glutamic acid at position 681, also located on the negative strand and resulting in a missense mutation in exon 19 (Figure 6A). Glutamine is a polar uncharged (neutral) amino acid. In contrast, glutamic acid is a polar, negatively charged residue. The Gln-to-Glu substitutions (and vice versa) are common benign variants in many proteins, almost like a synonymous missense change. However, since the variant (p.Gln681Glu) is located in the C-terminal domain of the TRIM37 protein, a change from Gln to Glu could possibly disrupt the protein’s ability to bind to the target proteins for ultimate ubiquitination (Figure 6B). This could interfere with the normal regulation of other cellular proteins, resulting in numerous complex developmental defects. We can then deduce that the synergistic effect of p.Arg67Cys and p.Gln681Glu may have resulted in major changes in the structure and function of the TRIM37 protein, leading to the severe clinical phenotype observed in our proband. However, further confirmation studies are needed to confirm the causality. Importantly, even though analysis showed that the proband was heterozygous for both [c.199C>T: (p.Arg67Cys)] and [c.2041C>G: p.Gln681Glu variant] (Table 1), the only inherited the c.199C>T: p.Arg67Cys variant from the mother, who is an immigrant. Unfortunately, the proband’s father was not involved and no paternal genetic information was provided that could facilitate the interpretation of the variants seen in our patient. Therefore, it’s unclear whether the second mutation was de novo or inherited from the father leading to compound heterozygous mutations. However, given the frequency of de novo mutations in *TRIM37* is very rare in this type of dwarfism, it is most likely that the second mutation did come from the father, and the two variants are in trans, leading to a compound pattern of inheritance.

Hämäläinen et al. 2004, reported the missense mutation [c.965G>T: (p.Gly322Val] in exon 12 after examining seven individuals with Mulibrey nanism who were not of Finnish descent. This was the first known mutation that was linked with Mulibrey nanism [18]. The pathogenicity of the novel variant was established by the fact that it was in the TRAF domain of the TRIM37 protein, causing a change in the subcellular localization of the mutant TRIM37 protein. Among six other mutations in that cohort, five caused a truncated protein due to genomic loss (8.6 kb) [18]. The most common *TRIM37* mutations associated with various heart disorders reported are frameshift, nonsense mutations, splice site variations, and stop codons that result in the early termination of the protein product along its complete length, as well as at the N-terminus (Table 3 ) [7,19,27,28]. The main reason for the termination is attributed to the lack of protein synthesis or the formation of a truncated protein lacking crucial functional domains [6–8]. With time, intragenic rearrangements and total gene deletion have also been identified, which may be a result of several repeated Alu elements in the genomic region [6,8]. As of 2020, more than 20 TRIM37 mutations have been reported after its first discovery, and they all been confirmed to be associated with Mulibrey nanism [5–8,23,28–30] (Table 3). Based on data from the Genome Aggregation Database (gnomAD), the *TRIM37* gene is generally considered to be intolerant to missense variation. While specific, up-to-the-minute Z-scores for every single transcript can fluctuate between gnomAD versions (v2.1.1 to v4.0), genes associated with severe, recessive disorders like Mulibrey nanism (caused by *TRIM37* loss-of-function) often exhibit high constraint metrics (Z-score ≥3.09).

## Limitations and Conclusions

Compared to the typical clinical features of Mulibrey nanism, the patient described in this report had more severe clinical manifestations and several congenital heart defects. To our knowledge, there is no publication related to this phenotypic variant in the literature to date. Also, it is noteworthy that the clinical and genetic features of Mulibrey nanism are quite diverse and may intersect and overlap with other conditions [6].

Considering that marriage among relatives raises the risk of rare autosomal recessive disorders, we could not confirm this with our patient. Exome sequencing has proven to be relevant to reliably identify two novel variants in our proband. But the absence of trio exome sequencing prevented it from being able to ascertain if the variants inherited by the proband were potentially a compound heterozygote variant.

We did not perform further analysis to ascertain the effects of these novel variants on the TRIM and the C-terminal domain; however, observations from our study highlight that the severity of the disease differs extensively. Hence, prioritizing antenatal care would have provided early detection, timely interventions, and subsequent management. There is therefore a need for intensive research to increase our knowledge of the underlying mechanism and pave the way to improve patient care and quality of life.

## Figures and Tables

**Table 1.** Summary of Proband WES results: Two novel heterozygous variants in *TRIM37* were identified.

Gene & Transcript	Genomic Position (hg19)	Nucleotide change	Protein change	Prediction	Location	Zygosity	Disorder (OMIM)	Inheritance
*TRIM37* NM_015294.3	chr17: 57165734	C.199C>T	p.Arg67Cys	VUS	Exon 4	Heterozygous	Mulibery nanim (MIM:253250)	Mother
*TRIM37* NM_015294.3	chr17: 57105992	C.2041C>G	p.Gln681Glu	VUS	Exon 19	Heterozygous	Mulibery nanim (MIM:253250)	Unknown (not in mother)

**Table 2:** Summary of Reported TRIM37 mutations associated with heart disease

Variant	Associated heart disease	Region affected Type of variant	References
c. 199C>T p.Arg67Cys	Mesocardia, interrupted arch type B, left sided superior vena-cava, multivalvular disease	Exon 4 Missense B-box domain	Current study
c.2041>G p.Gln681Glu	Mesocardia, interrupted arch type B, left sided superior vena-cava, multivalvular disease	Exon 19 Missense TRAF domain	Current study
c.493-2A>G	Constrictive pericarditis, myocardial hypertrophy, myocardial fibrosis	3’splice site Exon 7	Avela et el., 2000
c.221delG	Progressive congestive heart failure, right-sided heart disease	Deletion Exon 19	Avela et el., 2000
c.1949-12A>G	Diastolic impairement, constrictive pericardiatis	Splice-site Intron 18/ Exon 19	Mozzillo et al., 2016
c.370-IG>A	Progressive cardiomyopathy, peripheral edema	Splice-site	-
c.181>T p.Arg61*	Congestive heart failure	Nonsense Exon 4	Jobic et al., 2017
c.1411C>T p.Arg471X	Cardiomyopathy	Nonsense Exon 15	Hämäläinen et al., 2004
c.326G>C p.Cys109Ser	Cardiomyopathy	Missense Exon 5 B-box domain	Hämäläinen et al., 2004
c.965G>T p.Gly322Val	Cardiomyopathy	Missense Exon 12 MATH domain	Hämäläinen et al., 2004

**Table 3:** Summary of TRIM37 Mutations described in the literature and their consequences.

Number	Nucleotide change	Protein change	Age at diagnosis Sex	Mode of Inheritance	Predicted Consequence	Cause of death & Age at death	Exon	Origin	References
1	c.199C>T	p.Arg67Cys	32 weeks male	heterozygous	Missense	Cardiac arrest	4	Egypt	Current study
2	c.2041C>G	p.Gln681Glu	32 weeks male	-	Missense	Cardiac arrest	19	-	Current study
3	c.855_862del TGAATTAG	-	-	-	-	-	-	Turkish	-
4	c.81delG	-	-	-	Deletion	-	2	French	Fokkkema et al., 2011
5	c.181C>T	p.Arg61Ter	-	mother	Nonsense/stop codon	-	4	French	Jobic et al., 2017
6	c.181C>T	p.Arg61Ter	-	-	-	-	15-16 9 deletion	-	Jobic et al., 2017
7	c.227T>C	-	-	-	Missense	-	4	Finnish	Kalljarvi et al., 2005
8	c..326G>C	-	-	-	Missense	-	5	Austrian	Hämäläinen et al., 2004
9	c.493-2A>G	-	-	-	Splice-site	-	7	Finnish	Avela et al., 2000
10	c.685_809del	p.Leu299*	-	-	Large deletion	-	9	French	Jobic et al., 2017
11	c.745C>T	p.Gln249X	4yrs female	heterozygous	Nonsense	38yrs Congestive heart failure	9	Canadian	Hämäläinen et al., 2004
12	C.810-1G>A	-	-	-	Splice-site	-	10	Turkish	Jagieollo., et al 2003
13	c.838-842delACTTT	-	-	-	Deletion	-	10	Czech	Avela et al., 2000
14	c.860G>A	-	-	-	Splice-site	-	10	Australian	Hämäläinen et al., 2004
15	c.874_877delAGAG	-	-	-	Deletion	-	11	Japanese	Yasuhara et al., 2018
16	c.965G>T	p.Gly322Val		heterozygous	Missense	-	12	Canadian	Hämäläinen et al., 2004
17	c.1016C>G	-	-	-	Missense		13	Japanese	Yasuhara et al., 2018
18	c.1037_1040dup AGAT	p.Met347fsX7	4yrs female	heterozygous	Duplication (framshift at 347)	38yrs Congestive heart failure	13	Canadian	Hämäläinen et al., 2004
19	c.1166A>G	-	-	-	Nonsense	-	13	Finnish	-
20	c.1233delA	-	-	-	Deletion	-	14	German	Kumpf et al., 2013
21	c.1313+507_1668-207del	p.Arg439fsX4	-	-	Large Deletion	--	15,16	Italian	Hämäläinen et al., 2004
22	c.1314+507_1668-207del	p.Arg439fsX4	4yrs male	homozygous	-	Alive	15,16	Sicilian	Hämäläinen et al., 2004
23	c.1315_1667del	p.Arg439Valfs*5	-	father	Large Deletion	-	15-16	French	Jobic et al., 2017
24	c.1346dulA	-	-	-	Duplication	-	15	American	Avela et al., 2000
25	c.1411C>T	p.Arg471X	12yrs female	homozygous	Nonsense	Alive	15	Tunisian, German	Hämäläinen et al., 2004
26	c.1411C>T	p.Arg471X	- female	heterozygous	-	4yrs Renal and heart failure	15	Canadian	Hämäläinen et al., 2004
27	c.1894_1895delGA		-		Deletion	-	18	Turkish	Doganci et al., 2007
28	c.1910_1911dupTA	--	-		Duplication	-	18	Swiss	Fokkkema et al., 2011
29	c.1949-12A>G	-	-		Splice-site		19	Italian	Mozzillo et al.. 2016
30	c.2056C>T	p.Arg686X	6yrs male	homozygous	Nonsense	Alive	19	Saudi-Arabian	Hämäläinen et al., 2004
31	c.2212delG	-	-	-	Deletion	-	19	Finnish	Avela et al., 2000
32	c.1949-12A>G	-	-	-	Splice-site	-	19	Iatlian	Mozzillo et al., 2016

## Data Availability

The datasets used and analyzed during the current study are available from the corresponding author upon reasonable request. The study was registered in dbGaP under Novel Gene-Environment Regulatory Circuit in Chamber-Specific Growth of Perinatal Heart, Study ID: 45333. The stable dbGaP accession for this study is phs002725.v1. p1.

## Abbreviations:

ACMG: American College of Medical Genetics and Genomics
CHD: Congenital Heart Defect
CNV: Copy Number Variant
C-terminal: Carboxyl-terminal
DES: aspartate-glutamate-serine
FISH: Florescence in situ hybridization
HGMD: Human Gene Mutation Database
MN: Mulibrey nanism
NLS: Nuclear Localization Sequence
N-terminal: Amino-terminal
OMIM: Online Mendelian Inheritance in Man
PBMC: Peripheral blood monocytes
PDA: Patent Ductus Arteriosus
PGE: Prostalglandin E
RBCC: Ring-B-box-Coiled-coil
RV: Right ventricle
SNPs: Single nucleotide polymorphisms
SVC: Superior vena cava
TRAF: tumor necrosis factor-receptor-associated factor
TRAF: Tumor necrosis factor-receptor-associated factor
TRIM: Tripartite motif
UCLA: University of California Los Angeles
VSD: Ventricular septal defect
VUS: Variant of uncertain significance
WES: Whole-exome sequencing
